# MoBPSweb: A web-based framework to simulate and compare breeding programs

**DOI:** 10.1093/g3journal/jkab023

**Published:** 2021-02-04

**Authors:** Torsten Pook, Lisa Büttgen, Amudha Ganesan, Ngoc-Thuy Ha, Henner Simianer

**Affiliations:** Center for Integrated Breeding Research, Department of Animal Sciences, Animal Breeding and Genetics Group, University of Goettingen, Goettingen D 37075, Germany

**Keywords:** MoBPS, breeding, program, resource, management, simulation, population, genetics, nginx

## Abstract

In this study, we introduce a new web-based simulation framework (“MoBPSweb”) that combines a unified language to describe breeding programs with the simulation software MoBPS, standing for “Modular Breeding Program Simulator.” Thereby, MoBPSweb provides a flexible environment to log, simulate, evaluate, and compare breeding programs. Inputs can be provided via modules ranging from a Vis.js-based environment for “drawing” the breeding program to a variety of modules to provide phenotype information, economic parameters, and other relevant information. Similarly, results of the simulation study can be extracted and compared to other scenarios via output modules (*e.g.*, observed phenotypes, the accuracy of breeding value estimation, inbreeding rates), while all simulations and downstream analysis are executed in the highly efficient R-package MoBPS.

## Introduction

Since early prehistory, selective breeding has been a tool that humanity has used to, among others, ensure the food supply with examples spanning back to the development from teosinte to maize in Mesoamerica and animal breeding for war horses and dogs in the Roman Empire (Sidnell 2007). Over time, breeding has become more refined with concepts like genetic inheritance (Mendel 1866), quantitative genetics (Galton 1889; Fisher 1918) and population genetics (Wright 1922) being introduced. Today, breeders have a large toolbox of procedures and methods at their disposal, ranging from high-throughput genotyping and phenotyping (Solberg *et al.* 2006; Cabrera-Bosquet *et al.* 2012) to highly advanced biotechnology (Jinek *et al.* 2012) to complex quantitative models for breeding value estimation and QTL detection (Meuwissen *et al.* 2001; Klein *et al.* 2005; VanRaden 2008). These advanced methods are both a blessing and a curse, as managing and optimizing such a breeding program is a highly complex problem (Henryon *et al.* 2014).

In recent years a variety of tools to simulate breeding programs (Sargolzaei and Schenkel 2009; Faux *et al.* 2016; Liu *et al.* 2019; Pérez-Enciso *et al.* 2020; Pook *et al.* 2020) have been developed to aid breeders in their management decisions as they provide a controlled and repeatable environment to modify individual parameters of a breeding scheme and by that draw conclusions on their impact on the breeding objective. Potential selection goals usually include traits such as productivity, fitness, adaptation, and inbreeding. In contrast to deterministic analysis approaches like ZPLAN+ (Täubert *et al.* 2010), the mentioned simulators use stochastic simulations, thus allowing to draw conclusions on the variance of outcomes and provide more flexibility in the design of the breeding program.

A common problem of software to perform stochastic simulations is that they are complex to set up, as sound knowledge of available parameters and functions is required [*e.g.*, the main function of MoBPS (Pook *et al.* 2020) *breeding.diploid()* has over 200 parameters]. This leads to available features not easily being found or used incorrectly by potential users. Thus, simulation studies are oftentimes carried out via new and self-written code that is tailor-made for the problem at hand. However, this leads to the problem of inefficient and error-prone code that potentially neglects less-intuitive but still important factors that nonetheless affect the breeding scheme.

In a companion paper, Simianer *et al.* (2020) proposed a unifying concept to describe breeding programs. Even though the focus in that work was mostly on animal breeding, concepts can readily be extended to plant breeding. The key idea of the concept introduced by Simianer *et al.* (2020) is to describe each breeding program via a set of nodes and edges, with nodes representing cohorts of individuals and edges representing a set of potential breeding actions (*e.g.*, generation of offspring, performing phenotyping or selection of individuals). By this, Simianer *et al.* (2020) are providing a comprehensive, unambiguous, and reproducible way to describe a breeding program.

In this study, we combine the ideas of Simianer *et al.* (2020) with the highly efficient breeding program simulator MoBPS (Pook *et al.* 2020) to provide a web-based application (“MoBPSweb”) to log, simulate, evaluate, and compare breeding programs in an user-friendly and intuitive web-based environment.

## Materials and methods

**Figure 1:**
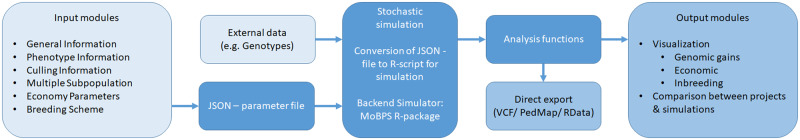
Schematic overview of the MoBPSweb framework.

MoBPSweb is a web-based application running on a NodeJS server that uses the VueJS and ExpressJS frameworks in the frontend and backend, respectively. We further employ MongoDB as a backend database (Chodorow 2013) and provide access to the application via nginx. The simulation of the breeding program and all downstream analyses are performed in R (R Core Team 2021) by using wrapper functions of the R-package MoBPS. An overview of the modules contained in the framework is given in Figure 1 with all sub-modules being described in more detail in the following subsections.

### Breeding scheme

As described in Simianer *et al.* (2020) a breeding scheme can be represented by a set of cohorts (nodes) and breeding actions (edges). This is implemented in an interactive environment based on the dynamic and browser-based visualization library Vis.js. To detect potential issues (*e.g.*, loops, missing required information), the resulting set of nodes and edges is constantly checked and potential issues are reported as warnings.

### Input modules

In addition to the breeding scheme itself, we provide a variety of different input modules to enter further information:

- General Information
- Phenotype Information
- Culling Information
- Multiple Subpopulations
- Economy Parameters

Except for the “General Information module,” these modules are optional and can be activated based on the need for the respective study. The basic functionality of each respective input module is described in the following subsections.

### General information

In this module, basic information like the species and the underlying genomic map (including used arrays) can be chosen. Maps can either be uploaded, manually created, or imported from the Ensembl database (Zerbino *et al.* 2018). Furthermore, smaller sub-modules are provided to control optional and very specific parameters such as the litter size, computational parameters that are mainly relevant for simulations with a high number of generations as in population genetic studies or to scale the size of each cohort (number of individuals) to reduce computational load and thus test the conformity of the breeding program on a smaller scale.

### Phenotype information

In this module, the underlying set of traits and their architectures can be defined. This includes defining phenotypic mean and variance, as well as the heritability, repeatability, and the number of underlying QTL for each trait. Potential correlations between traits need to be given for both the genetic and residual components. Furthermore, selection indices to later perform selection based on multiple traits can be defined here. Exemplary sets of traits for a variety of species are provided as templates.

### Culling information

In this module, rules for individuals leaving the breeding nucleus can be defined. In contrast to breeding actions via edges, no new individuals are generated. Input parameters are in line with parameters of the culling options in *breeding.diploid()* in the MoBPS R-package with the added benefit that the age of individuals is tracked and culling actions are automatically performed as soon as an individual reaches a given age.

### Multiple subpopulations

In this module, rules to generate founder individuals of different genetic origins can be defined, *e.g.*, for representing different breeds or lines in a crossbreeding scenario. This is only relevant in case no genotype information is imported for some of the founders and will lead to simulated genotypes being drawn from different per marker allele frequencies.

### Economy parameters

In this module, basic information regarding the cost of different breeding actions can be entered. This includes both fixed and variable costs like genotyping, phenotyping, and housing costs that are automatically discounted according to provided interest rates.

### JSON to R conversion

The output of the web-interface is a JavaScript Object Notation (JSON)-file containing all entered information of the breeding scheme. Subsequently, this JSON-file is translated into interpretable R-code. All this is implemented in the function *json.simulation()* in the R-package MoBPS (Pook *et al.* 2020). The translation procedure for all advanced modules is relatively straight-forward, as most parameters directly correspond to a parameter in the MoBPS R-package. For example, the size of the genome and the selected underlying array will be set as the map parameter in *creating.diploid()* and the phenotyping module is feeding in information for a separate call of *creating.trait()* from the MoBPS R-package.

The conversion of the breeding scheme itself is done by first detecting if the breeding scheme has any “Repeat” edges (Simianer *et al.* 2020), which are used to indicate that a given part of the breeding program is carried out multiple times (breeding cycles). If that is the case, it will subsequently check which nodes can be generated without the use of any repeat. Next, all repeats that can be executed based on the already available nodes are executed by generating copies of all nodes between the node of origin and the target node of the repeat (including the node of origin and excluding the target node). Nodes generated via repeat are serial-numbered via “_1,” “_2” etc. to indicate the repeat number. This procedure is repeated until all repeat edges are resolved, leading to a breeding program without any repeats remaining.

Next, the actual breeding scheme is simulated by first generating all founder nodes via *creating.diploid()*. All remaining cohorts are generated by separate calls of *breeding.diploid()*. All necessary information for this is stored in incoming edges and the node itself. For edges, this includes the breeding type, but also respective subsequent details (*e.g.*, for selection: the method for breeding value estimation, cohorts used for breeding value estimation, and so on.). For nodes, information includes the phenotyping class, the share of genotyped individuals, and the housing class. The order of generation will be derived based on generation times assigned to each edge and cross-dependencies (*e.g.*, when phenotyping information from one cohort is needed to generate breeding values for another cohort).

### Evaluating simulation outputs

After successfully simulating a breeding program, the resulting population-list is stored and can be analyzed. In the web-interface itself, we provide five implemented analysis modules, each corresponding to an analysis function from the MoBPS R-package:

- Observed Phenotypes—*get.pheno()*
- True Genomic Values—*get.bv()*
- Accuracy of Breeding Value Estimation—*analyze.bv()*
- Relationship and Inbreeding within Cohorts—*kinship.emp.fast()*
- Major QTLs—*get.geno()*

All these modules can either be applied on a single run of the simulation or be averaged across multiple random replicates of the same simulation scenario. Furthermore, the “Compare Project module” is providing an environment to compare different breeding schemes with each other by use of the same analysis functions.

Note that it is also possible to directly extract pedigree and phenotype information, VCF (Danecek *et al.* 2011)/PedMap (Purcell *et al.* 2007)-files for selected cohorts or the population-list itself (.RData) to proceed with own analyses in R.

## Results and discussion

In the following, we will discuss an exemplary use case of the web-interface for a dairy cattle breeding program on farm level. A variety of other breeding schemes that can be simulated via MoBPSweb including a commercial layer breeding program [Supplemental File S2, (Simianer *et al.* 2020)], the inclusion of health traits in horse breeding [Supplemental File S3, (Büttgen *et al.* 2020)], a cock rotation to preserve genetic diversity in chicken (Supplemental File S4) and the generation of a MAGIC population in maize (Supplemental File S5) are given as templates at www.mobps.de.

### Baseline scenario

**Figure 2:**
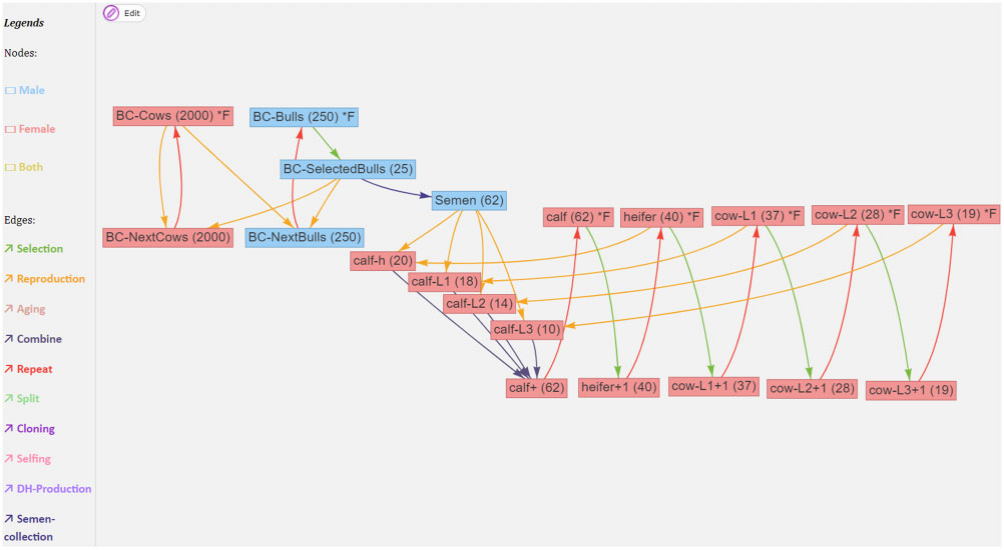
A dairy cattle selection program within a herd, with animals separated into age groups. Details on the attributes of all nodes and edges can be found in Supplementary Material S1.

The breeding scheme for the dairy cattle breeding scheme, as entered in the flash application of MoBPSweb, is given in Figure 2.

At each time point, the farm has spots for 184 animals consisting of five cohorts (calf, heifer, cow-L1, cow-L2, and cow-L3) that are split based on age. New animals are generated by the use of semen from a breeding company with heifers and older cows being used for reproduction. These offspring are then merged into a joined cohort and subsequently used as the calf-cohort (“calf+”; Figure 2) in the next repeat. New animals for the other four cohorts are chosen by selecting the required number of animals from the respectively 1 year younger cohort (“heifer+1,” “cow-L1+1,” “cow-L2+1,” “cow-L3+1”; Figure 2). For this selection procedure, five traits are simulated and animals are selected based on a selection index formed from those traits (Table 1), with phenotypes only being partially available based on age. It is assumed that already combined traits, as being used in the standard German breeding evaluation system are measured on each cow. The five traits RZM (milk), RZE (type), RZR (fertility), RZS (somatic cell score), RZKm (calving traits) were chosen according to Vereinigte Informations systeme Tierhaltung v.W (2020) and standardized to have a starting mean of 100 with a genetic standard deviation (gSD) of 12 to allow a better comparative assessment. Genetic correlations were taken from Vereinigte Informations systeme Tierhaltung v.W (2020) and residual correlations are assumed to be the same (Table 2). For simplicity reasons, longevity traits are ignored here as phenotyping of those traits can be more complex to model. Since longevity (RZN) with an index weight of 20 was removed from the analysis, the index weights of the remaining traits add up to 80. As traits given here already represent combinations of other traits, no heritability or repeatability values are given in Vereinigte Informations systeme Tierhaltung v.W (2020). Instead, reasonable values were estimated based on the given sub-trait heritabilities and using estimates in the literature (Roman *et al.* 2000; Oyama *et al.* 2002).

**Table 1:** Overview of the simulated traits for heritability, repeatability, index weights, and whether traits are phenotyped for each cohort (Vereinigte Informations system Tierhaltung w. V. 2020). Numbers in brackets indicate the accumulated number of observations for the respective trait.

Trait	Heritability	Repeatability	Index weighting	Calf	Heifer	Cow-L1	Cow-L2	Cow-L3
**RZM (milk)**	0.30	0.50	45	No	No	Yes (1)	Yes (2)	Yes (3)
**RZE (type)**	0.25	0.25	15	No	No	Yes (1)	Yes (1)	Yes (1)
**RZR (fertility)**	0.02	0.06	10	No	Yes (1)	Yes (2)	Yes (3)	Yes (4)
**RZS (somatic cell score)**	0.15	0.22	7	No	No	Yes (1)	Yes (2)	Yes (3)
**RZKm (calving traits)**	0.05	0.09	3	No	No	Yes (1)	Yes (2)	Yes (3)

**Table 2:** Genetic/residual correlation for considered traits given in Table 1 in the lower/upper triangle matrix.

Trait	RZM	RZE	RZR	RZS	RZKm
**RZM (milk)**	1	0.00	−0.25	−0.05	−0.05
**RZE (type)**	0.00	1	0.10	0.20	0.00
**RZR (fertility)**	−0.25	0.10	1	0.20	0.25
**RZS (somatic cell score)**	−0.05	0.20	0.20	1	0.10
**RZKm (calving traits)**	−0.05	0.00	0.25	0.10	1

**Figure 3:**
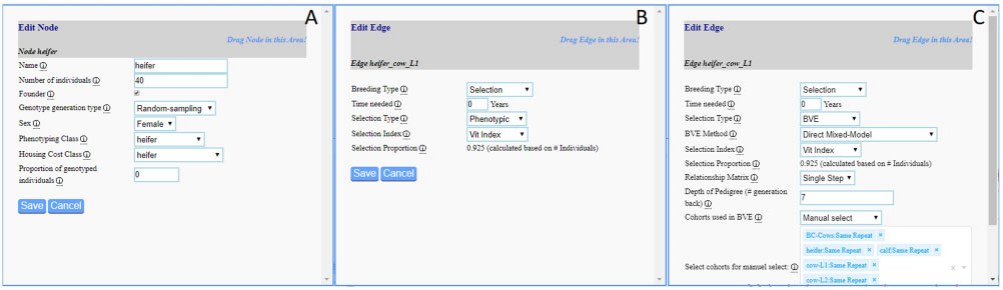
Exemplary node (A) and edge (B) of the baseline scenario (Figure 2) and the genomic selection edge used in the scenario “ssBLUP_BVE” (C).

In the baseline scenario, the selection on the farm is assumed to be solely based on individual phenotypes and paternal breeding material is received in the form of semen from the breeding company. The underlying breeding scheme from the side of the breeding company is simplified with just one male and one female cohort per cycle and small animal numbers (250 bulls, 2,000 cows) as it is not the main focus of this study. Selection for the bulls is done via single-step breeding value estimation (Aguilar *et al.* 2010; Christensen and Lund 2010) with only bulls being genotyped and phenotypes of the last two repeats of cows being used. Cows of the breeding company are assumed to have the same number of phenotypic observations per trait as cows after the third lactation period (“cow-L3,” Table 1). The breeding cycle was repeated 20 times with additional five burn-in repeats to build up some linkage disequilibrium and obtain an initial pedigree structure. An exemplary node and edges from the web interface are given in Figure 3, with details on all edges and nodes given in Supplemental File S1.

**Figure 4:**
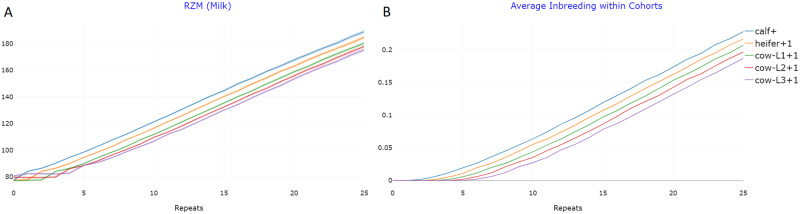
Genetic gain for the trait RZM (milk) with average genomic value standardized to 100 after 5 repeats (A) and increase in inbreeding (B) in the reference dairy cattle breeding scheme (Figure 2) with 95% confidence bands. Note that as deviations are extremely low, confidence bands are virtually invisible. These figures are exemplary outputs of the “True Breeding Values” and “Relationship and Inbreeding within Cohorts” modules in MoBPSweb (www.mobps.de).

The results of the simulation predict that the selection program achieves sustained gains with the highest increase for RZM (7.4 gSD in 20 repeats, Figure 4A). The corresponding results for the other traits are given in Supplementary Figures S1–S4. In addition, inbreeding rates increased by about 0.01 per cycle (Figure 4B). Prediction accuracies for the breeding value estimation of the bulls were relatively low with values ranging between 0.44 for RZR and 0.58 for RZE (which could easily be increased by enlarging the training population).

### Comparative scenarios

In addition to the baseline scenario, we will consider a variety of modifications of the original breeding program and analyze their impact:

1. Use of pedigree-based breeding value estimation for the selection of cows to be kept in the next cycle (“pedigree_BVE”)
2. Genotyping of the calves and subsequent selection via single-step BLUP on the farm (“ssBLUP_BVE”)
3. Reducing the selection intensity in the breeding company by 50% (Top 50 instead of Top 25 sires; “Low_SelectionIntensity”)
4. Modification of the selection index to put less weight on milk gain (RZM: 30, RZE: 20, RZR: 15, RZS: 10, RZKm: 5) (“Change_IndexWeights”)

**Figure 5:**
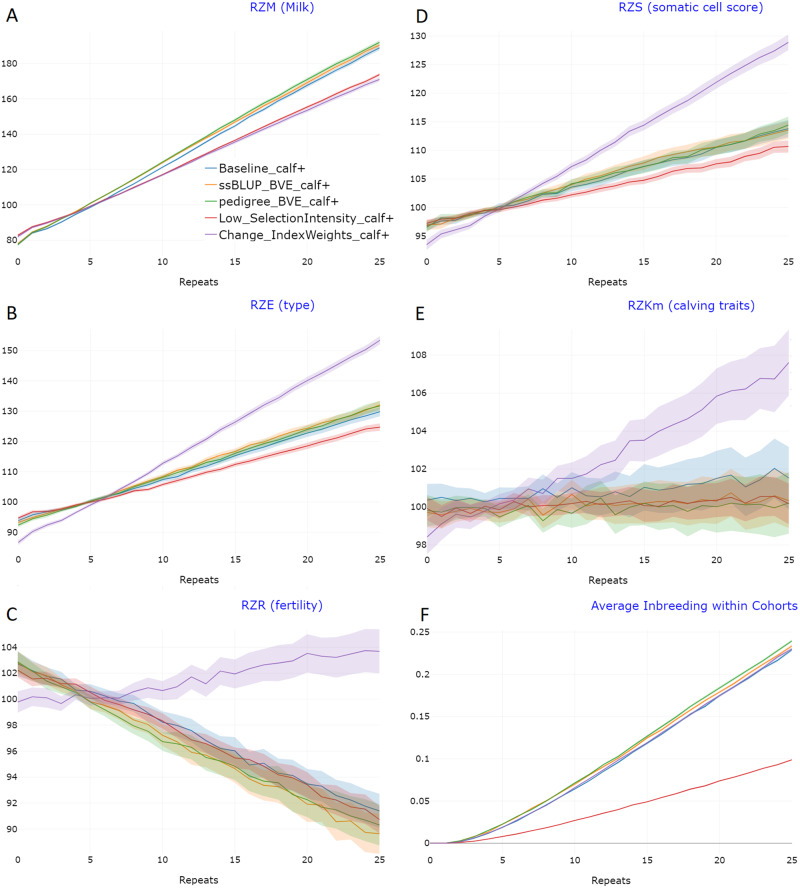
Genetic gain and the increase in inbreeding for the different scenarios of the cattle breeding program with 95% confidence bands for the traits RZM (A), RZE (B), RZR (C), RZS (D), RZKm (E), and inbreeding rates (F). Genomic values for all traits were standardized to an average of 100 after 5 repeats. This figure is an exemplary output of the “Compare Project module” in MoBPSweb (www.mobps.de).

To achieve reliable results, all scenarios were simulated 100 times and reported results represent averages across these runs. JSON-files of all scenarios can be found in Supplementary File S1. An overview of the development in the different scenarios for the traits and the rates of inbreeding are given in Figure 5. By the use of a breeding value estimation (“pedigree_BVE”/ “ssBLUP_BVE”) all traits can be considered for selection, even when no phenotypes are available for the respective cohort. Even though both scenarios led to a statistically significant increase in RZM (*t*-test, *p* = 0.0096/0.033, Figure 5A), the practical differences between scenarios were small, as most of the genetic gain generated on the male side of the breeding program. The prediction accuracies achieved by both prediction methods were relatively low (*e.g.*, for RZM around 0.42 for calves, Supplementary Figure S5). To increase the effectiveness of such intra-herd selection, one could further consider to select only half of the cows to produce heifers, while the other half could be mated to beef bulls to produce fattening calves. Note that this would require the use of sexed semen.

The reduction of the selection intensity on the side of the breeding company by 50% led to a 57% reduction of the inbreeding rates, and thus resulted in inbreeding rates of less than 0.005 per repeat (Figure 5F). This however came at the cost of a 20% reduction of genetic progress for RZM (Table 3, Figure 5A). However, the ratio between genetic gain and inbreeding is highest in this scenario. Note that when considering a longer time horizon, genetic progress in this scenario might even be highest as genetic gains in other scenarios will potentially diminish due to lower remaining genetic diversity. To analyze this in detail, more breeding cycles would have to be simulated. Furthermore, potential countermeasures like the introduction of new diversity into the breeding nucleus should be considered then.

**Table 3:** Average genomic values and rates of inbreeding after 25 cycles of breeding for the newly generated calves with genomic values being standardized to 100 after 5 cycles. Numbers in brackets indicate the estimated standard error of the obtained averages.

Scenario	RZM (milk)	RZE (type)	RZR (fertility)	RZS (somatic cell score)	RZKm (calving traits)	Inbreeding
**Baseline**	188.9 (0.70)	129.8 (0.77)	91.4 (0.69)	113.8 (0.76)	101.5 (0.83)	0.229 (0.00082)
**pedigree_BVE**	192.0 (0.62)	131.7 (0.84)	90.3 (0.80)	114.4 (0.75)	100.2 (0.83)	0.239 (0.00087)
**ssBLUP_BVE**	190.4 (0.65)	132.1 (0.70)	89.6 (0.80)	113.5 (0.77)	100.3 (0.73)	0.234 (0.00090)
**Low_SelectionIntensity**	173.7 (0.45)	124.7 (0.63)	90.7 (0.54)	110.7 (0.55)	100.2 (0.55)	0.099 (0.00035)
**Change_IndexWeights**	171.0 (0.67)	153.5 (0.71)	103.7 (0.87)	128.9 (0.69)	107.6 (0.89)	0.230 (0.00079)

Modification of the selection index to put less weight on the RZM led to a 22% smaller increase in this trait (Table 3). However, for all other traits performance was best, as this was the only scenario where an improvement for RZR was obtained and genetic gains for RZS were doubled (Figure 5, C and D).

Computing times for each simulation were about 20.5 minutes with about 15 minutes used for breeding value estimation, 4 minutes to generate new animals, and 30 seconds for both JSON-to-R conversion and initialization of the founder population using a single core of an Intel(R) Xeon(R) Gold 6132 CPU 2.60 GHz.

## Discussion

MoBPSweb provides an interactive, flexible, and efficient platform to simulate, evaluate, and compare breeding programs. Simulation studies are a valuable tool for breeders to quantify the effects of their chosen breeding actions, to complement their experience and quantitative genetics theory (*e.g.*, expected gains via the breeders’ equation). This is particularly relevant, as breeding programs are complex by nature and breeding actions are interconnected. Examples of this are lower rates of inbreeding when switching from pedigree-based to genomic breeding values (de Roos *et al.* 2011) or the loss of genetic diversity when increasing the selection intensity. By the use of a simulation study, a variety of output variables of a breeding program can be analyzed jointly, allowing to select the best solution for achieving a given breeding objective and thus optimize the breeding program. This solution will of course be highly dependent on the breeding objective, the general framework, and potential auxiliary conditions.

More fundamentally, the concepts introduced in Simianer *et al.* (2020) and the input environment given via MoBPSweb are providing a standardized, unambiguous, and reproducible way to describe breeding programs and by this are solving common problems of unclear terminology for breeding programs. Thereby, the described framework can also be seen as a management tool for breeding programs in general and will be useful for planning costs, time, and resources required.

With this, MoBPSweb makes the execution of complex simulation studies more accessible to researchers, breeders, and students and thus provides a platform to be used in teaching. In general, we would highly encourage other researchers to make their software programs more accessible via similar web-based applications and thus make their tools available to potential users who are less familiar with the respective back-end programming language.

All simulations in MoBPSweb can be executed without the use of R for the user. However, in case the output modules are not sufficient to extract the exact information one is interested in, it is also possible to download the resulting population-list and perform manual analyses in R. A further potential reason for the use of the R-package is the overall higher flexibility in the design of the breeding program, for instance, when considering complex phenotypes like longevity in cattle or when working with sophisticated selection schemes that are using new and/or own methodology.

Although the MoBPSweb interface does not provide additional functionality compared to the direct use of the R-package, it still offers major advantages in terms of use, as available parameter settings are provided more naturally and the order of breeding actions (*e.g.*, which cohort to generate when, when to generate phenotypes or individuals leading the breeding nucleus due to age/culling) is automatically taken care of. This in turn reduces potential error sources when setting up a simulation study. For reference, we implemented the simulation of the baseline breeding program via the R-package directly, which required about 200 lines of code (Supplemental File S5), while ignoring economic tracking and performing no downstream analysis. As the complexity of the breeding program increases in practice (*e.g.*, more nodes, aging and complex breeding value estimations), R-scripts can quickly become much longer and more complex. Note that although MoBPSweb was created for the MoBPS R-package, in principle any stochastic simulator for breeding programs should be usable to perform the required back-end simulations.

Finally, note that no simulation study will be able to fully capture reality in its entirety. Nevertheless, the key strength of simulation approaches lies in the fact that, in contrast to real-world experiments and field trials, far less time and money are needed to carry them out and potential harm to animals, such as adverse fitness effects, are avoided. Furthermore, experiments can be repeated and modified without any practical issues, which leads to much higher statistical power when comparing scenarios. Even if the absolute size of estimated effects might be off due to simplifications of reality, these effects should usually affect all considered scenarios and thus still ensure comparability between scenarios.

### Web resources

All code underlying MoBPSweb can be found at https://github.com/tpook92/MoBPS_web. A running version of MoBPSweb can be found at www.mobps.de. JSON-files for all compared scenarios discussed can be found in Supplemental File S1 with the reference also being included as a template at www.mobps.de. Supplemental Files S2, S3, S4, S5 provide JSON-file for other exemplary breeding programs, including a commercial layer breeding program (S2), the inclusion of health traits in horse breeding (S3), a cock rotation to preserve genetic diversity in chicken (S4), and the generation of a MAGIC population in maize (S5). Supplementary File S6 is providing exemplary R-code to simulate the presented baseline scenario. Supplementary Figures S1–S4 display the genetic gain for the traits RZR, RZE, RZS, RZKm for the reference dairy cattle breeding scheme. Supplementary Figure S5 provides information on the accuracy of the breeding value estimation for the different scenarios. Supplementary files are available at FigShare: https://doi.org/10.25387/g3.13573505.

## Funding

The MoBPS framework was developed in the context of the European Union’s Horizon 2020 Research and Innovation Program under grant agreement n °677353 IMAGE. We acknowledge support by the Open Access Publication Funds of the Göttingen University.

##  

*Competing Interests*: The authors declare no competing interest. The funders did not have any role in the study design, data collection and analysis, decision to publish, or preparation of the manuscript.

## References

[jkab023-B1] Aguilar I , Misztal I, Johnson DL, Legarra A, Tsuruta S, et al 2010. Hot topic: a unified approach to utilize phenotypic, full pedigree, and genomic information for genetic evaluation of Holstein final score. J Dairy Sci. 93:743–752. 20105546 10.3168/jds.2009-2730

[jkab023-B2] Büttgen L , Geibel J, Simianer H, Pook T. 2020. Simulation study on the integration of health traits in horse breeding programs. Animals 10:1153. 10.3390/ani10071153 PMC7401664 32645982

[jkab023-B3] Cabrera-Bosquet L , Crossa J, von Zitzewitz J, Serret MD, Luis Araus J. 2012. High–throughput phenotyping and genomic selection. J Int Plant Biol. 54:312–320. 10.1111/j.1744-7909.2012.01116.x 22420640

[jkab023-B4] Chodorow K. 2013. MongoDB: The Definitive Guide: powerful and Scalable Data Storage. “O’Reilly Media, Inc.” Sebastopol, California, USA.

[jkab023-B5] Christensen OF , Lund MS. 2010. Genomic prediction when some animals are not genotyped. Genet Sel Evol. 42:2. 20105297 10.1186/1297-9686-42-2 PMC2834608

[jkab023-B6] Danecek P , Auton A, Abecasis G, Albers CA, Banks E, et al 2011. The variant call format and VCFtools. Bioinformatics 27:2156–2158. 21653522 10.1093/bioinformatics/btr330 PMC3137218

[jkab023-B7] de Roos AP , Schrooten C, Veerkamp RF, van Arendonk JA. 2011. Effects of genomic selection on genetic improvement, inbreeding, and merit of young versus proven bulls. J Dairy Sci. 94:1559–1567. 21338821 10.3168/jds.2010-3354

[jkab023-B8] Faux A-M , Gorjanc G, Gaynor RC, Battagin M, Edwards SM, et al 2016. AlphaSim: software for breeding program simulation. Plant Genome 9:doi:10.3835/plantgenome2016.02.0013. 10.3835/plantgenome2016.02.0013 27902803

[jkab023-B9] Fisher RA. 1918. XV. - The correlation between relatives on the supposition of Mendelian inheritance. Trans R Soc Edinb. 52:399–433.

[jkab023-B10] Galton F. 1889. Natural Inheritance. London: Macmillan and Company.

[jkab023-B11] Henryon M , Berg P, Sørensen AC. 2014. Animal-breeding schemes using genomic information need breeding plans designed to maximise long-term genetic gains. Livestock Sci. 166:38–47.

[jkab023-B12] Jinek M , Chylinski K, Fonfara I, Hauer M, Doudna JA, et al 2012. A programmable dual-RNA–guided DNA endonuclease in adaptive bacterial immunity. Science 337:816–821. 22745249 10.1126/science.1225829 PMC6286148

[jkab023-B13] Klein RJ , Zeiss C, Chew EY, Tsai J-Y, Sackler RS, et al 2005. Complement factor H polymorphism in age-related macular degeneration. Science 308:385–389. 15761122 10.1126/science.1109557 PMC1512523

[jkab023-B14] Liu H , Tessema BB, Jensen J, Cericola F, Andersen JR, et al 2019. ADAM-plant: a software for stochastic simulations of plant breeding from molecular to phenotypic level and from simple selection to complex speed breeding programs. Front Plant Sci. 9:1926. 30687343 10.3389/fpls.2018.01926 PMC6333911

[jkab023-B15] Mendel G. 1866. Versuche über Pflanzen-Hybriden. Verhandlungen des. Naturforschenden Vereines in Brünn. 4:3–47.

[jkab023-B16] Meuwissen THE , Hayes BJ, Goddard ME. 2001. Prediction of total genetic value using genome-wide dense marker maps. Genetics 157:1819–1829. 11290733 10.1093/genetics/157.4.1819 PMC1461589

[jkab023-B17] Oyama K , Katsuta T, Anada K, Mukai F. 2002. Heritability and repeatability estimates for reproductive traits of Japanese Black cows. Asian Aust J Anim Sci. 15:1680–1685.

[jkab023-B18] Pérez-Enciso M , Ramírez-Ayala LC, Zingaretti LM. 2020. SeqBreed: a python tool to evaluate genomic prediction in complex scenarios. Genet Sel Evol. 52:9. 32039696 10.1186/s12711-020-0530-2 PMC7008576

[jkab023-B19] Pook T , Schlather M, Simianer H. 2020. MoBPS - Modular Breeding Program Simulator. G3: Genes, Genomes, Genetics 10:1915–1918. 32229505 10.1534/g3.120.401193 PMC7263682

[jkab023-B20] Purcell S , Neale B, Todd-Brown K, Thomas L, Ferreira MAR, et al 2007. PLINK: a tool set for whole-genome association and population-based linkage analyses. Am J Hum Genet. 81:559–575. 17701901 10.1086/519795 PMC1950838

[jkab023-B21] R Core Team, 2021. R: A Language and Environment for Statistical Computing. Vienna, Austria.

[jkab023-B22] Roman RM , Wilcox CJ, Martin FG. 2000. Estimates of repeatability and heritability of productive and reproductive traits in a herd of Jersey cattle. Genet Mol Biol. 23:113–119.

[jkab023-B23] Sargolzaei M , Schenkel FS. 2009. QMSim: a large-scale genome simulator for livestock. Bioinformatics 25:680–681. 19176551 10.1093/bioinformatics/btp045

[jkab023-B24] Sidnell P. 2007. Warhorse: Cavalry in Ancient Warfare. Bloomsbury Publishing.

[jkab023-B25] Simianer H , Ganesan A, Büttgen L, Ha NT, Pook T. 2020. A unifying concept of animal breeding programs. bioRxiv p. 2020.07.08.193201. 10.1111/jbg.12534 33486850

[jkab023-B26] Solberg LC , Valdar W, Gauguier D, Nunez G, Taylor A, et al 2006. A protocol for high-throughput phenotyping, suitable for quantitative trait analysis in mice. Mamm Genome 17:129–146. 16465593 10.1007/s00335-005-0112-1

[jkab023-B27] Täubert H , Reinhardt F, Simianer H. 2010. ZPLAN+, a new software to evaluate and optimize animal breeding programs. World Congress on Genetics Applied to Livestock. Leipzig, Germany p. 950.

[jkab023-B28] VanRaden PM. 2008. Efficient methods to compute genomic predictions. J Dairy Sci. 91:4414–4423. 18946147 10.3168/jds.2007-0980

[jkab023-B29] Vereinigte Informationssysteme Tierhaltung v.W. 2020. Estimation of Breeding Values for Milk Production Traits, Somatic Cell Score, Conformation, Productive Life and Reproduction Traits in German Dairy Cattle. Verden, Germany.

[jkab023-B30] Wright S. 1922. Coefficients of inbreeding and relationship. Am Nat. 56:330–338.

[jkab023-B31] Zerbino DR , Achuthan P, Akanni W, Amode MR, Barrell D, et al 2018. Ensembl 2018. Nucl Acids Res. 46:D754–D761. 29155950 10.1093/nar/gkx1098 PMC5753206

